# Amphetamine-type stimulants in young adults with ADHD: effects on sleep, circadian rhythms, and neuropsychiatric outcomes

**DOI:** 10.3389/fphar.2026.1891825

**Published:** 2026-08-07

**Authors:** Amelia Hanshaw, Alexandria Shanks, Alessandra Porcu

**Affiliations:** Department of Drug Discovery and Biomedical Sciences, College of Pharmacy, University of South Carolina, Columbia, SC, United States

**Keywords:** ADHD, amphetamine, chronotype, circadian rhythms, melatonin, psychosis, sleep, stimulants

## Abstract

Amphetamine-type stimulants are widely prescribed for the treatment of attention-deficit/hyperactivity disorder (ADHD) and are commonly used by college-aged individuals. While effective for improving attention and executive function, these medications may also influence sleep and circadian regulation. College students are particularly vulnerable to sleep disturbances because young adulthood is characterized by a biologically delayed circadian phase that favors later sleep timing. Combined with academic and social demands and nighttime light exposure, as well as the high prevalence of delayed circadian rhythms in ADHD, these factors may increase susceptibility to circadian misalignment and sleep disruption. This mini-review provides a narrative synthesis of the current literature examining the relationship between amphetamine-type stimulant use, sleep and circadian dysregulation, and neuropsychiatric outcomes in young adults with ADHD. Understanding how stimulant pharmacology interacts with the circadian system and sleep may help optimize treatment strategies and reduce side effects in young adults with ADHD.

## Introduction

Attention-deficit/hyperactivity disorder (ADHD) is a neurodevelopmental disorder characterized by hyperactivity, inattention, and impulsivity that typically begins in childhood but frequently persists into adulthood (Simon et al., 2009; Faraone et al., 2015). Worldwide, approximately 2%–5% of adults are estimated to suffer from ADHD (Simon et al., 2009; Song et al., 2021). In the United States alone, an estimated 15.5 million adults had received an ADHD diagnosis by 2023, with nearly half of these diagnoses occurring in adulthood (Centers for Disease Control and Prevention, 2025). Among these individuals, stimulant medications remain the primary pharmacological treatment, although barriers to medication access and evolving patterns of care, including increased reliance on telehealth since the COVID-19 pandemic, have influenced diagnosis and treatment trends (Cortese et al., 2018; Swanson et al., 2011).

Amphetamine-type stimulants are among the most prescribed medications for ADHD. These drugs increase synaptic concentrations of dopamine and norepinephrine by promoting neurotransmitter release and inhibiting reuptake (Sulzer et al., 2005; Heal et al., 2013). Commonly prescribed formulations include lisdexamfetamine, dextroamphetamine, and mixed amphetamine salts, which are typically administered once or twice daily depending on the formulation (Cortese et al., 2018; Childress, 2022). While these medications effectively improve attention and executive function, they also influence neuronal systems involved in arousal and sleep–wake regulation (Swanson et al., 2011; Volkow et al., 2011).

Sleep disturbances are among the most frequently reported adverse effects of stimulant medications (Snitselaar et al., 2017; Kidwell et al., 2015). Acute and chronic exposure to stimulants has been associated with delayed sleep onset, reduced total sleep time, increased nighttime awakenings, and alterations in sleep architecture, including reductions in slow-wave sleep and rapid eye movement sleep (Snitselaar et al., 2017; Kidwell et al., 2015; Spruyt and Gozal, 2011). These effects are particularly relevant in young adults, who exhibit a biologically delayed circadian phase that naturally favors later sleep and wake times. When combined with academic and social demands, nighttime light exposure, and the high prevalence of delayed circadian rhythms in individuals with ADHD, this developmental characteristic increases vulnerability to sleep disruption and circadian misalignment (Becker et al., 2018; Lund et al., 2010; Becker et al., 2024; Bijlenga et al., 2019; R et al., 2007).

In recent years, stimulant use and misuse have increased among individuals aged 18–23 years, with reports suggesting that approximately 14%–15% of college students have misused prescription stimulants (Arria et al., 2017; Benson et al., 2015; Clegg-Kraynok et al., 2011). Such misuse often involves higher doses or non-medical timing of administration to enhance academic performance (Arria et al., 2017; Benson et al., 2015). These patterns may further exacerbate sleep disruption and circadian misalignment, potentially contributing to adverse neuropsychiatric outcomes (Snitselaar et al., 2017; Kidwell et al., 2015; Becker et al., 2024).

**FIGURE 1:**
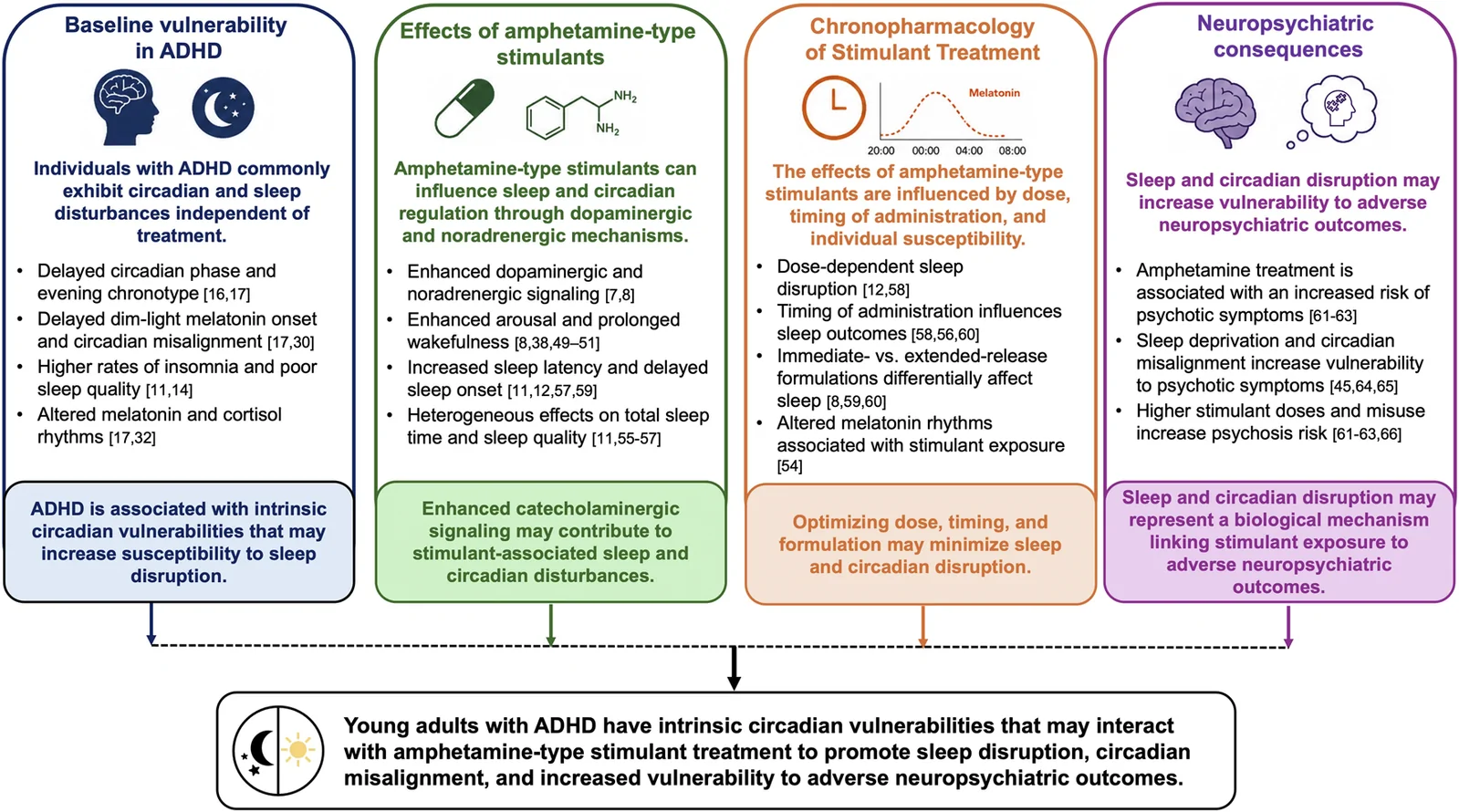
Proposed framework linking amphetamine-type stimulant treatment, sleep and circadian disruption, and neuropsychiatric outcomes in young adults with ADHD. Individuals with ADHD exhibit intrinsic sleep and circadian vulnerabilities, including delayed circadian phase, evening chronotype, sleep disturbances, and alterations in melatonin and cortisol rhythms. Amphetamine-type stimulants influence sleep and circadian regulation primarily through dopaminergic and noradrenergic mechanisms, although clinical responses are heterogeneous. The magnitude of these effects is further influenced by chronopharmacological factors, including dose, timing of administration, formulation, and individual susceptibility. Together, these interactions may increase vulnerability to adverse neuropsychiatric outcomes, particularly in young adults with ADHD, a population characterized by ongoing neurodevelopment and biologically delayed circadian timing.

Despite growing evidence linking stimulant use to sleep disturbances and mental health outcomes, relatively little is known about how amphetamine-type stimulants influence the circadian system in young adults with ADHD (Snitselaar et al., 2017; Becker et al., 2024). While previous reviews have examined sleep disturbances in ADHD and the effects of stimulant medications on sleep, few have integrated circadian mechanisms, stimulant pharmacology, and psychiatric outcomes within a single framework, particularly in young adult populations (Snitselaar et al., 2017; Kidwell et al., 2015; Becker et al., 2024). This mini-review provides a narrative synthesis of the current literature examining the relationship between amphetamine-type stimulant use, sleep and circadian dysregulation, and neuropsychiatric outcomes in young adults with ADHD (Figure 1). Relevant articles were identified through searches of PubMed and Google Scholar using combinations of the keywords ADHD, amphetamine, stimulants, sleep, circadian rhythms, chronotype, melatonin, psychosis, and young adults. Priority was given to primary research articles, systematic reviews, and meta-analyses published in English. Studies focusing on amphetamine-type stimulants in adolescents and young adults, as well as investigations of circadian mechanisms relevant to ADHD, were preferentially included. Studies primarily evaluating non-stimulant medications or not directly addressing sleep, circadian function, or neuropsychiatric outcomes were excluded.

## Role of the sleep and circadian system in mental health

Circadian rhythms are 24-h internal biological cycles regulated by the brain’s master clock in the suprachiasmatic nucleus (SCN), which synchronizes behavior and physiological processes to environmental light cues (Reppert and Weaver, 2002; Hastings et al., 2018; Takahashi, 2017). Disruptions to sleep and circadian timing are highly prevalent in individuals with ADHD, both in untreated populations and in those receiving stimulant medications (Snitselaar et al., 2017; Becker et al., 2024; Bijlenga et al., 2019; Coogan and McGowan, 2017; van Andel et al., 2021). Such disruptions, whether due to intrinsic circadian alterations, behavioral factors, or pharmacological influences, can impair critical physiological processes including memory consolidation, metabolic regulation, and emotional processing (Walker, 2009; Tononi and Cirelli, 2003; Van Cauter et al., 2008). Consistent with this, individuals with ADHD exhibit higher rates of sleep disturbances and are at increased risk for psychiatric comorbidities, including anxiety and mood disorders (Bijlenga et al., 2019; Coogan and McGowan, 2017; Katzman et al., 2017).

Environmental and behavioral cues, such as the timing of sleep, meals, and daily activities, play a key role in maintaining circadian alignment (R et al., 2007; Hastings et al., 2018; Takahashi, 2017). In young individuals with ADHD, these environmental cues often interact with an underlying predisposition toward delayed circadian timing, contributing to circadian misalignment and dysregulation of physiological rhythms such as cortisol secretion, core body temperature, and melatonin production (Bijlenga et al., 2019; R et al., 2007; Van der Heijden et al., 2005; Luu and Fabiano, 2025). In particular, delayed or reduced melatonin secretion has been reported in ADHD populations and may contribute to difficulties initiating sleep and maintaining stable sleep–wake cycles (Bijlenga et al., 2019; Van der Heijden et al., 2005; Baird et al., 2012; Crouse et al., 2018; van Andel et al., 2022). These alterations are further exacerbated by stimulant treatment, which increases arousal signaling and may delay sleep onset when administered later in the day (Snitselaar et al., 2017; Kidwell et al., 2015).

Collectively, these findings highlight the importance of sleep and circadian regulation in ADHD and suggest that both intrinsic circadian alterations and stimulant-related effects may contribute to sleep and circadian disturbances in this population (Snitselaar et al., 2017; Bijlenga et al., 2019; Coogan and McGowan, 2017). Given that young adults with ADHD are frequently exposed to irregular schedules, academic demands, and stimulant medications, understanding how circadian disruption emerges in this context is critical for identifying strategies to improve sleep and reduce vulnerability to adverse mental health outcomes.

## Young adulthood: a unique neurodevelopmental and circadian window

Young adulthood represents a unique developmental stage characterized by ongoing neurobiological maturation and distinct circadian physiology that may increase susceptibility to stimulant-induced sleep and circadian disturbances. Although adolescence encompasses the most rapid period of brain development, maturation of the prefrontal cortex and its associated executive control networks continues into the third decade of life through synaptic refinement and progressive myelination, supporting executive function, impulse control, emotional regulation, and reward processing (Fuhrmann et al., 2015; Larsen and Luna, 2018). These developmental processes are particularly relevant in individuals with ADHD, whose symptoms are closely linked to alterations in executive and reward-related neural circuits (Faraone et al., 2015). In parallel, mesocortical dopaminergic pathways continue to mature throughout adolescence and into early adulthood (Reynolds and Flores, 2021; Hoops and Flores, 2017), contributing to the refinement of executive function, reward processing, and cognitive control, processes that are directly targeted by amphetamine-type stimulants.

The circadian system also undergoes important developmental changes during this period. Circadian phase progressively delays throughout adolescence, reaching its latest timing during late adolescence and early adulthood before gradually advancing with increasing age (R et al., 2007; Logan and McClung, 2019; Crowley et al., 2007). Consequently, young adults exhibit a biological preference for later sleep and wake times, delayed dim-light melatonin onset (DLMO), and greater vulnerability to circadian misalignment when social and academic schedules require early awakening. This chronic mismatch between endogenous circadian timing and external demands, commonly referred to as social jetlag, contributes to sleep restriction and impaired cognitive and emotional functioning (R et al., 2007). Furthermore, circadian disruption during sensitive developmental periods has been increasingly recognized as an important contributor to brain dysfunction, influencing neural plasticity, reward processing, mood regulation, and psychiatric vulnerability across the lifespan (Logan and McClung, 2019). Together, these neurodevelopmental and circadian characteristics provide a strong biological rationale for focusing specifically on young adults, a population that may be particularly susceptible to the sleep- and circadian-related effects of stimulant treatment.

## Amphetamine-type stimulant effects on circadian regulation of dopaminergic and noradrenergic signaling

Amphetamine-type stimulants exert their primary pharmacological effects through interactions with the dopamine transporter (DAT), a membrane protein responsible for the reuptake of dopamine from the synaptic cleft (Sulzer et al., 2005; Heal et al., 2013; Vaughan and Foster, 2013). Under normal physiological conditions, DAT regulates extracellular dopamine concentrations by transporting dopamine back into presynaptic neurons, thereby terminating dopaminergic signaling and regulating attention, arousal and motivation (Sulzer et al., 2005; Vaughan and Foster, 2013; Kristensen et al., 2011). Amphetamines alter these processes by reversing DAT function, leading to the release of dopamine into the synaptic cleft and sustained increase in extracellular dopamine levels (Sulzer et al., 2005; Heal et al., 2013; Vaughan and Foster, 2013). Dopamine release from the ventral tegmental area (VTA) to the ventral striatum including the nucleus accumbens (NAc) exhibits circadian rhythmicity, with higher dopamine levels during the night phase in nocturnal rodents (Sleipness et al., 2007). Furthermore, dopamine synthesis, DAT, and dopamine receptor expression display circadian rhythmicity (Sleipness et al., 2007; Ferris et al., 2014; Porcu et al., 2020). Human imaging studies using positron emission tomography have demonstrated time-of-day variation in dopaminergic signaling, as reflected by changes in D2/D3 receptor binding that are sensitive to endogenous dopamine levels (Volkow et al., 2012). Additional evidence indicates that reward-related brain function exhibits robust circadian modulation, supporting a functional link between the circadian system and dopaminergic neurotransmission (Logan and McClung, 2019; Murray and Harvey, 2010; Hasler et al., 2010). By interfering with normal DAT-mediated dopamine clearance, amphetamine exposure may disrupt these circadian fluctuations in dopaminergic tone, leading to increased arousal signaling and delayed sleep onset (Heal et al., 2013; Vaughan and Foster, 2013; Vrajová et al., 2021). Such mechanisms may help explain how stimulant medications influence sleep architecture and circadian timing, particularly when taken at high doses or at inappropriate times of day.

In addition to increasing dopaminergic neurotransmission, amphetamine-type stimulants also enhance central noradrenergic signaling by promoting norepinephrine release and reversing norepinephrine transporter (NET) function (Sulzer et al., 2005; Heal et al., 2013). The noradrenergic system, originating primarily from neurons of the locus coeruleus (LC), plays a fundamental role in regulating arousal, attention, and sleep–wake transitions. LC neuronal activity is highest during wakefulness, decreases during non-rapid eye movement sleep, and is nearly absent during rapid eye movement sleep, highlighting its critical role in maintaining wakefulness (Van Egroo et al., 2022; Carter et al., 2010). Moreover, accumulating evidence indicates that LC activity is under circadian regulation through interactions with the suprachiasmatic nucleus (SCN), allowing circadian signals to modulate arousal across the day-night cycle (Logan and McClung, 2019; Aston-Jones et al., 2001). Consequently, amphetamine-induced enhancement of noradrenergic signaling may prolong wakefulness and delay sleep onset by amplifying endogenous arousal pathways, particularly when stimulants are administered later in the day.

Additionally, chronic amphetamine intake alters the expression of core clock genes in animal models; thus, medical use of amphetamine stimulant for ADHD might lead to further disruption of circadian rhythms, underlining the chronopharmacological importance of identifying the optimal timing of treatment (Heal et al., 2013; McClung, 2007). Together with altered dopaminergic and noradrenergic transmission, these mechanisms likely contribute to the sleep and circadian disturbances observed in young adults receiving amphetamine-type stimulants.

## Sleep and circadian disruptions associated with ADHD and stimulant use

Several studies have reported sleep and circadian disturbances in individuals with ADHD, particularly in those treated with stimulant medications (Snitselaar et al., 2017). A systematic review by Snitselaar et al. (2017) examined studies published between 1990 and 2011 investigating sleep and circadian characteristics in adults with ADHD. Across the included studies, both subjective and objective measures of sleep were assessed, including sleep onset latency (SOL), sleep maintenance, total sleep time (TST), and morning awakening patterns (Snitselaar et al., 2017). Subjectively, approximately 87% of individuals with ADHD reported difficulty initiating sleep, and about 72% reported symptoms consistent with sleep onset insomnia (Snitselaar et al., 2017). Objective actigraphy recordings across several studies confirmed longer SOL and increased nocturnal awakenings compared with control populations (Snitselaar et al., 2017; Spruyt and Gozal, 2011). Individuals with ADHD also exhibited shorter uninterrupted sleep bouts, greater sleep fragmentation, and later wake times, with one study reporting an average wake time approximately 46 min later than controls (Snitselaar et al., 2017; Spruyt and Gozal, 2011). Additionally, 70% of patients reported difficulty waking in the morning, suggesting broader circadian timing alterations in this population (Snitselaar et al., 2017).

Sleep disturbances associated with ADHD symptoms have also been observed in large university populations (Becker et al., 2018; Becker et al., 2024). Becker et al. (2018) investigated sleep problems in 7,626 college students aged 18–29 years using the Pittsburgh Sleep Quality Index (PSQI) alongside measures of ADHD symptoms (Becker et al., 2018). Inattentive ADHD symptoms were significantly associated with poorer sleep quality and greater daytime dysfunction, even after controlling for depression and anxiety symptoms (Becker et al., 2018). More recently, accumulating evidence has further characterized ADHD as a disorder frequently accompanied by circadian rhythm dysfunction, including delayed chronotype, DLMO, altered cortisol rhythms, and disrupted clock gene expression, all of which contribute to impaired sleep and daytime functioning (Bijlenga et al., 2019; Luu and Fabiano, 2025; Baird et al., 2012; Fargason et al., 2013).

Emerging evidence suggests that amphetamine-type stimulants may influence circadian regulation in individuals with ADHD (Snitselaar et al., 2017; Coogan and McGowan, 2017; Logan and McClung, 2019). A preliminary study in individuals with amphetamine dependence reported reduced serum melatonin concentrations compared with healthy controls, suggesting that chronic stimulant exposure may also disrupt circadian hormone regulation (Khazaie et al., 2019). Furthermore, observational studies have associated stimulant use with an evening chronotype and delayed sleep timing, including later mid-sleep on free days (Becker et al., 2024; Bijlenga et al., 2019; Coogan and McGowan, 2017). However, because these findings are largely derived from observational studies, it remains unclear whether they reflect direct effects of stimulant treatment or whether individuals with more severe ADHD symptoms and greater baseline circadian disruption are more likely to receive pharmacological therapy. Although causality cannot be inferred from these studies, the available evidence suggests that pharmacological effects and underlying circadian vulnerability may interact to influence sleep and circadian health outcomes in this population.

In contrast to the evidence discussed above, recent studies suggest considerable interindividual variability in sleep responses to stimulant treatment. Clinical trials in adults have reported that lisdexamfetamine does not significantly worsen overall sleep quality despite treatment-emergent reports of insomnia (Adler et al., 2009), while more recent longitudinal evidence suggests that stimulant treatment may even be associated with improved sleep quality and reduced odds of insomnia over time (Ada et al., 2026). These beneficial effects may reflect improved control of core ADHD symptoms, resulting in reduced bedtime hyperactivity, improved behavioral regulation, and more consistent sleep routines rather than direct sleep-promoting effects of the medication itself. Similarly, a recent study of adolescents with ADHD found that stimulant medication produced only small and inconsistent effects on objective and subjective sleep parameters (Wiggs et al., 2024). Although modest increases in sleep onset latency and later sleep onset were observed, stimulant treatment did not appear to be a primary contributor to sleep problems, highlighting substantial variability in sleep responses across individuals. However, comparable studies specifically examining young adults with ADHD remain limited, warranting further investigation in this population.

## Chronopharmacological effects of stimulant timing and formulation

The timing of stimulant exposure may also influence circadian regulation and sleep physiology (Heal et al., 2013; Coogan and McGowan, 2017; Logan and McClung, 2019). Serum melatonin rhythms have been shown to be altered in individuals with amphetamine dependence and circadian rhythm sleep disorders, with significantly lower melatonin concentrations at 00:00, 04:00, and 08:00 compared with healthy controls, suggesting that stimulant exposure may disrupt circadian hormone rhythms (Khazaie et al., 2019).

Research examining the relationship between stimulant timing and sleep outcomes has also reported dose- and timing-dependent effects on sleep quality (Kidwell et al., 2015; Herrmann et al., 2017). Although not meeting the strict inclusion criteria for the present review, one controlled study reported that morning administration of methamphetamine approximately 14 h before bedtime significantly disrupted nighttime sleep in a dose-dependent manner, as assessed using objective sleep measures (Herrmann et al., 2017). Higher stimulant doses were also associated with poorer sleep outcomes, supporting a dose-dependent relationship between stimulant exposure and sleep disruption (Kidwell et al., 2015; Herrmann et al., 2017).

The pharmacokinetic properties of stimulant formulations may also influence circadian timing and sleep outcomes. Immediate-release (IR) formulations have a shorter duration of action, whereas extended-release (ER) formulations maintain therapeutic plasma concentrations for a longer period, potentially prolonging catecholaminergic signaling into the afternoon or evening (Heal et al., 2013). Studies of extended-release methylphenidate have demonstrated increased sleep onset latency and reduced total sleep time in medication-naïve children, while sleep efficiency remained unchanged and slow-wave sleep was preserved (Corkum et al., 2020). These findings suggest that both formulation and dosing schedule should be considered when evaluating stimulant-related sleep disturbances. However, direct comparisons between immediate-release and extended-release amphetamine formulations on circadian phase markers, including DLMO and sleep architecture, remain limited, highlighting an important area for future investigation. Overall, current evidence suggests that sleep outcomes are influenced not only by stimulant formulation but also by dose and timing of administration, underscoring the importance of chronopharmacological considerations when prescribing stimulant medications (Heal et al., 2013; Kidwell et al., 2015; Ada et al., 2026; Corkum et al., 2020; Stein et al., 2022).

## Are sleep and circadian disruption potential mediators of stimulant-induced psychosis?

Evidence also suggests that stimulant exposure may be associated with an increased risk of psychotic symptoms (McKetin et al., 2019; Moran et al., 2019; Mesquita et al., 2022). Although this association is largely attributed to enhanced dopaminergic and noradrenergic neurotransmission (Sulzer et al., 2005; Heal et al., 2013), chronic sleep disruption and circadian misalignment may represent important biological mediators. Experimental and clinical studies have shown that prolonged sleep deprivation can impair emotional regulation, alter dopaminergic signaling, and induce psychosis-like symptoms, suggesting that stimulant-induced sleep loss may lower the threshold for psychosis in vulnerable individuals (Waters et al., 2018; Meyer et al., 2024).

A large cohort study by Moran et al. (2019) examined adolescents and young adults aged 13–25 years with ADHD who initiated treatment with either amphetamine or methylphenidate (Moran et al., 2019). Among 221,846 individuals included in the final analysis, the risk of new-onset psychosis was significantly higher in patients receiving amphetamine compared with those receiving methylphenidate (hazard ratio = 1.65, 95% CI 1.31–2.09) (Moran et al., 2019). Similarly, systematic reviews by McKetin et al. (2019) and Mesquita et al. (2022) reported that amphetamine use is associated with an increased risk of psychotic symptoms, with evidence supporting a dose-dependent relationship (McKetin et al., 2019; Mesquita et al., 2022).

Patterns of stimulant misuse and inappropriate dosing schedules may further increase this risk by exacerbating sleep disruption and circadian misalignment. Beckmann et al. (2020) described a case of stimulant-induced psychosis in a patient who repeatedly administered immediate-release amphetamine formulations throughout the day rather than following the prescribed extended-release regimen (Beckmann et al., 2020). Such dosing patterns may prolong wakefulness, increase cumulative sleep deprivation, and further disrupt circadian rhythms. Given that young adults with ADHD are already predisposed to delayed circadian timing and insufficient sleep, these factors may act synergistically with stimulant pharmacology to increase psychiatric vulnerability.

## Conclusion

Young adults with ADHD represent a particularly vulnerable population because intrinsic circadian abnormalities frequently coexist with a developmental delay in circadian phase and the widespread use of amphetamine-type stimulant medications. Current evidence suggests that amphetamine-type stimulants may further influence sleep and circadian regulation by enhancing dopaminergic and noradrenergic signaling, potentially exacerbating delayed sleep timing and circadian misalignment in susceptible individuals. Although the relationship between stimulant treatment and sleep outcomes is heterogeneous, growing evidence indicates that circadian disruption may represent an important biological mechanism contributing to adverse neuropsychiatric outcomes, including mood disturbances and psychotic symptoms, particularly in individuals exposed to higher doses or inappropriate patterns of stimulant use.

Despite increasing recognition of the interaction between stimulant pharmacology and the circadian system, important knowledge gaps remain. Future longitudinal studies should integrate objective measures of circadian function, including dim-light melatonin onset, actigraphy, and chronotype assessment, together with sleep quality, medication timing, and psychiatric outcomes in young adults with ADHD. A better understanding of these interactions may help optimize stimulant treatment, guide chronopharmacological approaches, and reduce the risk of sleep-related adverse effects while preserving the therapeutic benefits of these medications.
